# Decreased Iron Ion Concentrations in the Peripheral Blood Correlate with Coronary Atherosclerosis

**DOI:** 10.3390/nu14020319

**Published:** 2022-01-13

**Authors:** Heyu Meng, Yueying Wang, Jianjun Ruan, Yanqiu Chen, Xue Wang, Fengfeng Zhou, Fanbo Meng

**Affiliations:** 1 Jilin Provincial Precision Medicine Key Laboratory for Cardiovascular Genetic Diagnosis (Jilin Provincial Engineering Laboratory for Endothelial Function and Genetic Diagnosis of Cardiovascular Disease, Jilin Provincial Molecular Biology Research Center for Precision Medicine of Major Cardiovascular Disease, Jilin Provincial Cardiovascular Research Institute), Jilin University, Changchun 130033, China; hymeng20@mails.jlu.edu.cn (H.M.); ruanjj19@mails.jlu.edu.cn (J.R.); chenyq20@mails.jlu.edu.cn (Y.C.); xwang18@mails.jlu.edu.cn (X.W.); 2 Cancer Systems Biology Center, Jilin University, Changchun 130033, China; yueyingw21@mails.jlu.edu.cn (Y.W.); ffzhou@gmail.com (F.Z.); 3 BioKnow Health Informatics Lab, College of Computer Science and Technology and Key Laboratory of Symbolic Computation and Knowledge Engineering of Ministry of Education, Jilin University, Changchun 130012, China

**Keywords:** trace elements, iron ions, coronary atherosclerosis, acute myocardial infarction, stable coronary artery disease

## Abstract

(1) Background: Obesity and diabetes continue to reach epidemic levels in the population with major health impacts that include a significantly increased risk of coronary atherosclerosis. The imbalance of trace elements in the body caused by nutritional factors can lead to the progression of coronary atherosclerosis. (2) Methods: We measured the concentrations of sodium (Na), potassium (K), magnesium (Mg), calcium (Ca), Zinc (Zn), and iron (Fe) in peripheral blood samples from 4243 patients and performed baseline analysis and propensity matching of the patient datasets. The patients were grouped into acute myocardial infarction (AMI, 702 patients) and stable coronary heart disease (SCAD1, 253 patients) groups. Both of these groups were included in the AS that had a total of 1955 patients. The control group consisted of 2288 patients. The plasma concentrations of calcium, magnesium, and iron were measured using a colorimetric method. For comparison, 15 external quality assessment (EQA) samples were selected from the Clinical Laboratory Center of the Ministry of Health of China. SPSS software was used for statistical analysis. The average values and deviations of all of the indicators in each group were calculated, and a *p*-value threshold of <0.05 was used to indicate statistical significance. (3) Results: The iron ion concentrations of the acute myocardial infarction (AMI) group were significantly lower than the control group (*p* < 0.05, AUC = 0.724, AUC = 0.702), irrespective of tendency matching. Compared to the data from the stable coronary artery disease (SCAD) group, the concentration of iron ions in the acute myocardial infarction group was significantly lower (*p* < 0.05, AUC = 0.710, AUC = 0.682). Furthermore, the iron ion concentrations in the (AMI + SCAD) group were significantly lower (*p* < 0.05) than in the control group. (4) Conclusions: The data presented in this study strongly indicate that the concentration of iron ions in the peripheral blood is related to coronary atherosclerosis. Decreases in the levels of iron ions in the peripheral blood can be used as a predictive biomarker of coronary atherosclerosis.

## 1. Background

The levels of obesity and diabetes continue to increase around the world on an epidemic scale [1]. Between 2011 and 2012, more than one-third of the American population was clinically obese. Obesity and diabetes have become huge public health problems in the United States and around the world [2,3]. On a global scale, obesity is a more serious health problem than hunger, and it is the main cause of death and disability worldwide. The public health burden of obesity is expected to increase in the next few years [4]. The medical expenses of obese people are on average 42% higher than normal-weight patients, whilst the medical expenses of diabetic patients are about twice those of non-diabetic patients [1,5,6].

The World Health Organization (WHO) defines obesity as “abnormal or excessive fat accu mulation with health risks”. Hippocrates wrote that obesity is not only a disease, but also a precursor to other diseases [7]. Body mass index (BMI) has been widely used since it was proposed in 1972 [7]. BMI is divided into four categories, Class I (Underweight) < 18.5, Class II (Healthy): 18.5 to 24.9, Class III (Overweight): 25 to 29.9, and Class IV (Obese): 30+. Overweight and obesity have a major impact on the physical, psychological, and social health of individuals and have a negative impact on society in the form of increased medical expenditure [7,8]. Traditional diet, cereals, and low-fat, mixed dishes have been replaced by high-fat meat diets, while the intake of cereals and vegetables has been reduced, leading to increased intake of animal foods, processed foods, sugar-sweetened beverages, and processed foods high in fat, energy, sugar, and salt [9,10]. This shift has led to a major change in the macronutrient composition of the Chinese diet. The shift from a high-carbohydrate diet to a high-fat diet is accompanied by negative changes in health. Overweight and obesity have become a prominent problem for adults. The prevalence increased from 16.4% and 3.6% in 1982 to 30.1% and 11.9% in 2012 [11,12,13]. The contribution of fat-energy intake exceeded the recommended value of the Chinese Dietary Guidelines (CDG) and increased from 18.4% in 1982 to 32.9% in 2012 [14,15,16].

According to the China Cardiovascular Disease Report, 11 million patients suffer from coronary heart disease. The incidence of cardiovascular diseases in China is on the rise, accounting for more than 40% of disease deaths [17]. In 2016, the mortality rate of AMI in urban areas in China was 58.69/100,000, and the mortality rate of AMI in rural areas was 74.72/100,000 [18].

Obesity and diabetes are multifactorial, complex diseases that are also preventable in most cases [19,20] and significantly increase the risk of coronary atherosclerosis [21]. Atherosclerotic cardiovascular disease (ACD) is the leading cause of global mortality [5]. Over time, high body mass index (BMI), blood pressure, blood glucose, and cholesterol have different effects on the mortality rate in patient groups of different incomes [22,23]. In the past 20 years, high-income countries have been able to reduce the impact of these risk factors, whilst the mortality rate in low- and middle-income countries due to high BMI and hyperglycemia has increased [23]. The increase in mortality can be attributed to population growth and aging, as well as important changes in the dietary system [10]. It is not known whether differences in the dietary intake of trace elements contribute to the development of coronary atherosclerosis [24,25].

Research studies have shown that trace elements in the body are closely related to many disease including coronary heart diseases [26,27]. In a previous study from our lab, we found that zinc ions are related to menopause, and supplementation of zinc ions has a positive effect on the treatment of coronary heart disease [26]. Kalita H et al. confirmed that changes in the content of trace elements can improve insulin resistance caused by type 2 diabetes [28]. The study demonstrated that magnesium and manganese are cofactors of a variety of related enzymes, and low levels of these elements increase the risk of metabolic syndrome. Furthermore, Li et al. found that serum selenium levels are significantly related to the all-cause mortality of men and women but are more significant in women with coronary heart disease [29].

The purpose of this research was to explore the relationship between serum trace elements and coronary atherosclerosis. Based on big data, fasting peripheral blood samples of 4243 patients were collected to detect the levels of sodium, potassium, magnesium, calcium, and iron. The difference in serum iron concentration between different groups was analyzed to determine whether the serum iron level is a risk factor for coronary heart disease.

## 2. Research Subjects and Methods

### 2.1. Research Process and Sample

#### 2.1.1. Research Process

A total of 4243 patients who were hospitalized in the Department of Cardiology at The Third Hospital of Jilin University from October 2017 to December 2018 were included in the study. Amongst the cohort, 702 patients had an acute myocardial infarction, 1253 patients had stable coronary artery disease, and 2288 patients had non-coronary heart disease. The selected patients all complied with the ethical principles of the Declaration of Helsinki on the medical research of human subjects. All patients were recruited under written informed consent, and all patient data were anonymized. Detailed patient records including medical history, smoking history, and other relevant clinical data were recorded. Blood biochemical data including sodium, potassium, calcium, magnesium, zinc, iron, triglycerides, total cholesterol, high-density lipoprotein, and low-density lipoprotein levels were recorded.

Four groups of patients were defined in this study, specifically, the AMI, SCAD, coronary atherosclerosis (AMI + SCAD), and control groups. The diagnostic criteria for acute myocardial infarction met the European Society of Cardiology (ESC) diagnosis of acute myocardial infarction and were type I myocardial infarctions [30,31]. Stable coronary artery disease was diagnosed based on the ESC’s guidelines for coronary heart disease and met the stenosis of the main coronary artery at 50–75% (in most cases, the duration of short-term discomfort did not exceed 30 min).

The exclusion criteria for the coronary heart disease group were as follows: 1. myocardial infarction related to the percutaneous coronary intervention (PCI) or coronary artery bypass grafting (CABG), 2. type II myocardial infarction (secondary myocardial infarction) related to the blood supply and demand imbalance or myocardial infarction caused by elevated catecholamine levels or coronary artery spasm, 3. myocardial infarction accompanied by cardiac surgery or non-cardiac surgery.

In the selected control group, the diagnoses of AMI and SCAD were excluded. Arteriography showed that the stenosis of the main coronary artery and main branches was <50% with no corresponding symptoms.

The exclusion criteria for the control group were as follows: 1. multifactorial or uncertain myocardial damage caused by uncertain diseases (such as severe heart failure, stress cardiomyopathy, severe pulmonary embolism or pulmonary hypertension, sepsis, critical illness, or renal failure); 2. severe nervous system diseases including stroke and subarachnoid hemorrhage; 3. immune system diseases or hormone use; 4. history of tuberculosis (active or potential) or evidence of tuberculosis; 5. history of chronic or recurrent infectious diseases; 6. serious infectious diseases, complications of malignant tumors, or suspected or confirmed immunodeficiency.

**Figure 1:**
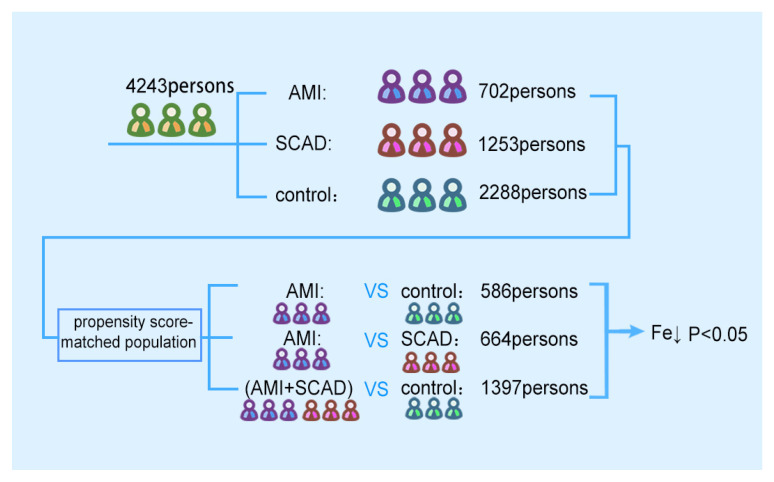
Flow chart of the study. The flow chart consists of the data before and after propensity matching. Iron ions were at low levels before and after propensity matching. AMI: acute myocardial infarction; SCAD: stable coronary artery disease.

The general flow chart for the work conducted in this study is shown in Figure 1. A total of 4243 patients were selected. The patients were divided into AMI, SCAD, and control according to the inclusion criteria. After propensity matching and analysis, the final conclusion was drawn.

#### 2.1.2. Subject Lifestyles and Patient Self-Reporting

Age, smoking, and diabetes were all self-reported by the patient and confirmed by their relatives. Blood glucose, triglycerides, total cholesterol, high-density lipoproteins, and low-density lipoproteins were tested in the Department of Laboratory Medicine, The Third Hospital of Jilin University. The study subjects were all from the same region and had similar eating habits and lifestyles. Generally speaking, breakfast is based on milk bread, lunch and dinner are based on vegetables and meat protein, and meat is based on pork. In this area, the pace of life is slow and the four seasons are distinct (from the patients’ self-reporting and objective situation).

### 2.2. Peripheral Blood Collection and Recording of Biochemical Indices

A morning 6 mL fasting peripheral blood was collected from each study participant in an EDTA anticoagulation tube and stored at 4 °C. The collected blood samples were delivered to the laboratory for biochemical analysis. The biochemical data and medical histories of the patients were analyzed.

### 2.3. Determination of Ion Concentrations

Detection kits for sodium, potassium, calcium, magnesium, zinc, and iron ions were used for analysis. A commercial assay kit for iron (Fe) ions (PAPS chromogenic reagent method, LEADMAN, Beijing) was used. Serum samples were stored at 2–8 °C and used for 2-point calibration with deionized water and an iron ion calibrator. The iron ions in the sample formed a colored complex with the nitro-PAPS in the reagent at room temperature, and the color of the solution is proportional to the concentration of iron ions. The accuracy and quality of the iron control were measured from 15 external quality assessment (EQA) samples selected from the Clinical Laboratory Center of the Ministry of Health. The concentration of zinc in each sample was measured in duplicate, and the average value was calculated. The qualified judgment standard was <1/2TEa (total allowable error of measuring iron concentration). Before the analysis, the samples were equilibrated at room temperature. The quality control software of the laboratory information system was used to measure the homogeneity of the quality control materials on the computer.

### 2.4. Statistical Analysis

The data were analyzed using SPSS 22.0 (IBM Corp., Armonk, NY, USA) statistical software. Quantitative data were expressed as quartile, and qualitative variables were expressed as the frequency. An independent Mann–Whitney U test was used to analyze the differences between continuous variables. A χ^2^ test was used to analyze the differences between categorical variables. The odds ratios (ORs) and 95% confidence intervals (95% CIs) were calculated. A two-tailed *p*-value < 0.05 was used to indicate statistical significance. Propensity score matching was performed using R software (version 3.6.1). In this study, we used propensity matching to analyze data. This matching method takes into account age, gender, smoking history, high blood pressure, diabetes, total cholesterol, triglycerides, high-density lipoproteins, and low-density lipoproteins. In the case of consistent baseline data, the experimental group and the control group are very different in age; this kind of age difference is likely to lead to inaccurate experimental results. In this study, in the description of baseline data, it is possible to use propensity matching analysis or multiple regression analysis. The main difference lies in the age difference between groups. Propensity matching analysis can balance the statistical information between groups well.

## 3. Results

### 3.1. Basic Data Analysis of Research Subjects

Data from a total of 4243 participants were analyzed in this study. The study cohort included 702 patients with myocardial infarction who had a median age of 68 years, 61.1% were male, and 53.4% were smokers. The stable coronary heart disease group included 1253 patients with a median age of 65 years, 52.4% were males, and 37.2% were smokers. The coronary atherosclerosis group included 1955 patients with a median age of 66 years, 55.5% were males, and 43.0% were smokers. The control group included 2288 cases with a median age of 56 years, 49.1% were males, and 32.2% were smokers. The data are summarized in Table 1, Table 2 and Table 3.

Differences in age, smoking history, diabetes or hypertension, total cholesterol level, and high-density lipoproteins were observed between the AMI, SCAD, and the control groups. Based on the above significant factors, the propensity score matching method was selected. After propensity matching, no significant differences in the distribution of the baseline characteristics were detected (data are shown in Table 1, Table 2 and Table 3). Elevated iron ion levels were identified as a common risk factor in the AMI, SCAD, and control groups (Table 4, Table 5 and Table 6).

Table 1 and Table 2 show the baseline analysis of different sets of data. Table 1 describes the baseline conditions of patients in the AMI group and the control group. Table 2 describes the baseline conditions of patients in the AMI group and SCAD group. Table 1, Table 2 and Table 3 are the baseline data of each group. Table 4, Table 5 and Table 6 are the experimental data corresponding to each group.

### 3.2. Comparison of the Acute Myocardial Infarction and Control Groups

Before propensity matching, 702 patients were in the acute myocardial infarction group, and 2288 patients were in the control group. Compared to the control group, the levels of calcium, magnesium, and iron ions were significantly different in the AMI group (*p* < 0.05). After propensity matching, 586 patients were in the experimental and the control groups, and the only significant difference between these groups was the level of iron ions (*p* < 0.05). These data were consistent with the trend before propensity matching which showed that the AMI group had higher levels of iron ions compared to the control group (Table 4).

### 3.3. Comparison of the Acute Myocardial Infarction and Stable Coronary Heart Disease Groups

Before propensity matching, there were 702 patients with acute myocardial infarction and 1253 patients in the stable coronary heart disease group. Compared to the SCAD group, the AMI group had significantly different levels of sodium, calcium, zinc, and iron ions (*p* < 0.05). After propensity matching, there were 664 patients in the experimental and SCAD groups which had significant differences in the levels of calcium, zinc, and iron ions (*p* < 0.05). These data were consistent with the trend before propensity matching which showed that the level of calcium, zinc, and iron ions in the AMI group were higher than in the SCAD group (Table 5).

### 3.4. Comparison of the Acute Myocardial Infarction and Stable Coronary Heart Disease Group with the Control Group

Before propensity matching, there were 702 patients with acute myocardial infarction and 1253 patients in the stable coronary heart disease group. Both AMI and SCAD are the result of coronary atherosclerosis, and the pathological changes are due to vascular stenosis caused by the increase in vascular plaque. Therefore, AMI and SCAD were mixed into the atherosclerosis group. Before propensity matching, there were 1955 cases in the atherosclerosis group and 2288 cases in the control group. Compared with the control group, the levels of calcium, magnesium, zinc, and iron ions were significantly different in the AS group (*p* < 0.05). After propensity matching, there were 1397 patients in the experimental and the control groups which had significantly different levels of iron ions (*p* < 0.05). These data were consistent with the trends before propensity matching that showed high levels of iron ions compared to the control group (Table 6).

### 3.5. ROC Analysis of the Trace Elements

**Figure 2:**
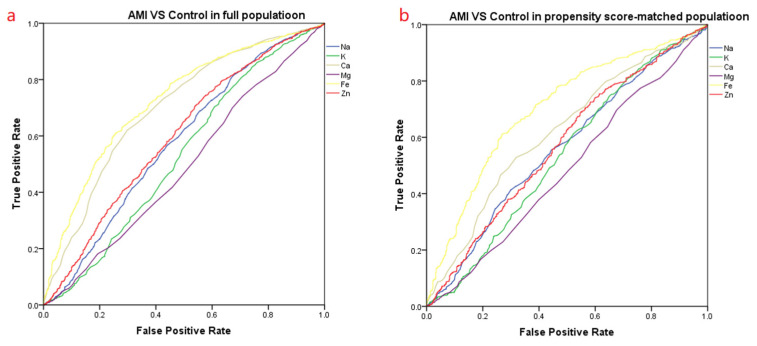
The ROC curve of the AMI vs. the Control group. (**a**)The ROC curve before propensity score-matching. (**b**) The ROC curve after propensity score-matching. The area under the curve of the various trace elements is summarized in Table 7.

**Figure 3:**
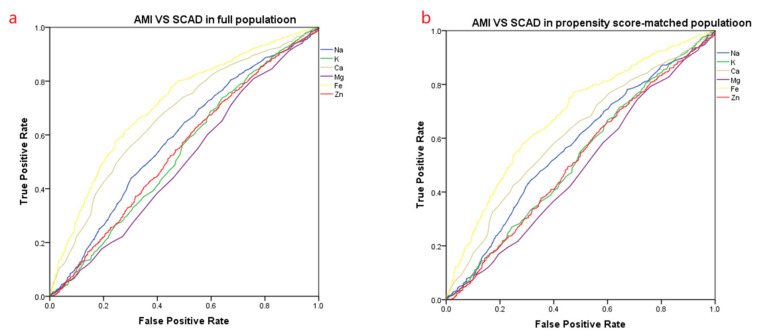
The ROC curve of the AMI vs. the SCAD. (**a**) The ROC curve before propensity score-matching. (**b**) The ROC curve after propensity score-matching. The area under the curve of the various trace elements is summarized in Table 8.

Before propensity matching, the difference in the area under the curve (AUC) of iron ions in the AMI group compared with the control group was 0.724. After propensity matching, the difference in the area under the curve of iron ions was 0.702 (Figure 2 and Table 7). Before propensity matching, the difference in the area under the curve (AUC) of iron ions in the AMI group compared with the SCAD group was 0.710. After propensity matching, the area under the curve of iron ions was 0.682 (Figure 3 and Table 8).

## 4. Discussion

Iron is an element that is essential for several physiological processes and plays important roles in cell metabolism through iron-containing proteins and enzymes. These proteins and enzymes maintain mitochondrial function, DNA synthesis and repair, and cell growth and death [32,33,34]. Iron is the main component of hemoglobin that is essential for the production of red blood cells and oxygen transport. However, iron may also be toxic at high concentrations as it can generate reactive oxygen species (ROS) and oxidize biomolecules through toxic hydroxyl radicals generated by the Fenton reaction [35]. Iron is also a key factor in determining the toxicity of bacteria [36].

Iron homeostasis is strictly controlled by the interactions of various iron processing tissues and cells (including macrophages, red blood cells, hepatocytes, and duodenal epithelial cells) and is regulated by the hepcidin–ferroportin axis. Most of the required iron is recovered from aging red blood cells by the reticulum–endothelial system. The extra demand for iron is fine-tuned by adjusting the amount of iron absorbed by the intestinal cells.

Dysregulated iron intake or output can cause illness. Iron is involved in the pathogenesis of atherosclerotic coronary heart disease (CAD) which was first proposed by Sullivan [35,37]. Free iron can accelerate the oxidation of low density lipoproteins (LDLs). Low density lipoproteins are then absorbed by low-density lipoprotein receptors on macrophages, which leads to the recruitment of foam cells. Foam cell infiltration and necrotic core expansion are key events in the formation of coronary atherosclerosis [38]. Macrophages play a central role in the progression of coronary atherosclerosis, and different subtypes of macrophages have been detected in atherosclerotic plaques [39]. Lipid uptake is the main stimulating factor for the differentiation of M1 macrophages in plaques which can induce the production of inflammatory cytokines and the formation of foam cells [40]. M1 macrophages can also induce SMC proliferation and migration from the media to the intima through paracrine actions and are considered to promote coronary atherosclerosis.

MMP-1, MMP-3, and MMP-9 released by M1 cells can hydrolyze collagen fibers in the fibrous cap which may lead to plaque instability [41]. Conversely, M2 macrophages are stimulated by Th2-type cytokines (such as IL-10, IL-4, and IL-13) to produce anti-inflammatory cytokines. M2 macrophages are thought to balance the inflammatory response to promote the resolution of inflammation and tissue repair. The M1/M2 paradigm provides a simplified framework to understand the function of macrophages in an injury environment. M1 macrophages are rich in ferritin and are prone to iron accumulation, whilst M2 macrophages can metabolize and export iron. The turnover rate of iron in M1 and M2 macrophages is different and can result in the development of coronary atherosclerosis.

In organisms, iron is usually combined with biomolecules such as heme resulting in an iron-deficient environment in the body. Bacteria must adapt to this environment through a series of iron acquisition mechanisms [36]. Siderophores are involved in these processes and have a high affinity for iron ions and can capture iron from protein-iron complexes [42]. Another way of ingesting iron relies on direct contact between the pathogen and the iron source [43,44]. AS is an inflammatory disease and has a higher risk of bacterial infection compared to patients without AS. Patients with AS present increased opportunities for bacteria to use iron which is supported by the observations of a decreasing trend in iron in this study.

γ-glutamyl transpeptidase (γ-GT) is present in serum and the plasma membrane of almost all cells. γ-GT initiates the hydrolysis of extracellular glutathione (GSH). GSH is a tripeptide in which cysteine is located between α-glycine and γ-glutamic acid residues. It is known that cysteine and other thiol compounds promote LDL oxidation by reducing Fe(III) to redox-active Fe(II). This process requires the consumption of trace iron in AS [45]. Therefore, it is feasible that patients with AS have lower concentrations of iron ions.

Magnesium (Mg) is a cofactor for various enzymes related to diabetes. Low levels of magnesium and manganese increase the risk of metabolic syndrome, which affects glucose metabolism. The lack of magnesium ions can lead to increased levels of total glycerides and total cholesterol, leading to coronary atherosclerosis [46,47]. Zinc (Zn) acts as a membrane stabilizer in vascular endothelial cells and participates in numerous signal transduction pathways in the cell, preventing the cell structure from being damaged by unstable factors such as oxidized lipids [48,49]. At the same time, there is a zinc regulation pathway in the blood vessel wall, which is combined with the production and effect of NO and is destroyed when zinc deficiency occurs, thereby affecting the onset and treatment of vascular diseases [50]. Serum calcium (Ca) levels are strictly regulated by parathyroid hormone and vitamin D. The imbalance of calcium ion in the patient’s body leads to secondary hyperparathyroidism, which in turn leads to the occurrence of coronary atherosclerosis [51].

IMT is a noninvasive marker of the severity of carotid atherosclerosis. There is no final conclusion on whether iron ion concentration changes arterial intima-media thickness [52], and some studies have suggested that iron ion concentration has no relationship with IMT [53]. ABI index can reflect the degree of lower extremity arterial stenosis and is an index to identify peripheral atherosclerosis. An increasing number of studies have linked ABI with coronary atherosclerosis, but its clinical value still needs to be judged. A 2019 study found that ABI can detect carotid atherosclerosis in healthy residents. It can prevent and help patients who have been identified as healthy [54].

There may be two main ways in which iron ions affect the occurrence of atherosclerosis. First, iron and hydrogen peroxide can oxidize a variety of matrices and cause biological damage. This reaction is called Fenton reaction. It is a complex reaction that can produce hydroxyl radicals and a higher oxidation state of iron [55]. The combined iron can prevent the catalytic reaction of iron producing free radicals [56]. Reactive oxygen species play a role in the development of cardiovascular disease by oxidizing low density lipoproteins (LDLs), which damage vascular endothelial cells. This process is not only the pathological mechanism of atherosclerosis but also the reason for the increase in arterial intima-media thickness. The second is inflammatory reaction. A randomized crossover study from different countries showed significant changes in postprandial oxidative stress, which may trigger inflammation and lead to endothelial dysfunction and atherosclerosis [57]. Iron ions are different in diets of different cultures. At the same time, the inflammatory process may also be improved by some nutrients (such as vitamin D, Omega 3 fatty acids [58,59] and tryptophan [60,61]) or exacerbated by metabolites (such as cysteine) [62]. Therefore, iron ions can promote the formation of free radicals or increase inflammatory response and establish a relationship with IMT and ABI.

At first, serum amyloid *p* substance was recognized as a kind of amyloid deposition protein, and it is believed that it plays an important role in the occurrence and development of systemic amyloidosis. Studies have gradually found that SAP participates in various activities such as innate immunity, inflammatory response, and amyloidosis in the body [63,64,65]. In recent years, the role of serum amyloid *p* in the cardiovascular system has gradually been recognized [66].

Isoprostaglandin is a stable metabolite of arachidonic acid. In normal human plasma and urine, its concentration is at least an order of magnitude higher than that of prostaglandins [67]. The concentration of isoprostaglandins, especially 8-iso-PGF_2α_, which is the most widely studied, increases under many pathophysiological conditions, including diabetes [68], Alzheimer’s disease [69], and allergic reactions [70]. Whether they can be used as disease markers, however, is largely unknown. The potency of 8-iso-PGE_2_ on 8-iso-PGF_2α_ and 8-iso-PGE_1_ on 8-isoPGF_1α_ indicates that the E-ring compound is more potent than the F-ring compound. Secondly, 8-iso-PGE_2_ is better than 8-iso-PGE_1_, and 8-iso-PGF_2α_ is better than 8-isoPGF_1α_, indicating that double-unsaturated compounds are stronger than mono-unsaturated compounds. Third, 8-iso-PGF_2α_ is much more potent than 8-iso-PGF_2β_, which shows the importance of α configuration [71]. This suggests that 8-iso-PGF_1α_ seems to be a partial agonist. Isoprostaglandins participate in the dynamic changes of atherosclerosis, which include vasodilation and contraction and platelet activation and inhibition [72,73]. This dynamic balance is the key to the stability of the cardiovascular system. This also suggests that follow-up studies can focus on the relationship between iron ions and isoprostaglandins.

There are many risk factors associated with coronary atherosclerosis including high blood pressure, diabetes, and smoking. Most of these risk factors can be modified through a healthy lifestyle or drugs [74]. For example, eating foods rich in fiber can significantly reduce the risk of coronary atherosclerosis [75]. Currently, coronary atherosclerosis is diagnosed based on multi-factor joint analysis [76] that can be enhanced by assessing the roles of specific trace elements. The optimized intake of trace elements in the diet can reduce the occurrence and improve the prognosis of coronary atherosclerosis.

There are some limitations in this article. This study is a retrospective analysis. ABI and IMT are classic markers of peripheral vascular atherosclerosis and carotid atherosclerosis respectively. Increasing this part of indicators can extend coronary atherosclerosis to systemic atherosclerosis. In addition, it may be more interesting to increase relevant indicators of oxidative stress and study the relationship between trace elements and oxidative stress.

## 5. Conclusions

In this study, acute myocardial infarction and coronary atherosclerosis were associated with lower expression levels of plasma iron ions. These data support the use of iron ions as an additional biomarker and prognostic indicator of coronary atherosclerosis.

## Figures and Tables

**Table 1:** Baseline characteristics and AMI-Controls risk factors in the study population.

	Full Population			Propensity-Score-Matched Population		
	AMI Patients (N = 702)	Controls (N = 2288)	*p*	AMI Patients (N = 586)	Controls (N = 586)	*p*
Age (years)	68.00(60.00,77.00)	56.00(47.00,66.00)	<0.001	67.00(59.00,74.00)	66.00(58.00,73.00)	0.553
Gender			<0.001			0.183
Male (%)	429(61.10%)	1124(49.10%)		359(61.30%)	381(65.00%)	
Female (%)	273(38.90%)	1164(50.90%)		227(38.70%)	205(35.00%)	
Smokers (Yes/No)	375/327	736/1552	<0.001	304/282	319/267	0.380
Hypertension (Yes/No)	361/341	994/1294	<0.001	292/294	298/288	0.726
Diabetes (Yes/No)	183/519	166/2122	<0.001	113/473	105/481	0.548
TG (mmol/L)	1.50(1.09,2.11)	1.35(0.96,2.02)	<0.001	1.50(1.08,2.12)	1.37(1.01,2.11)	0.117
Tc (mmol/L)	4.53(3.87,5.36)	4.58(3.91,5.31)	0.46	4.57(3.94,5.40)	4.55(3.87,5.31)	0.363
HDL (mmol/L)	1.02(0.88,1.21)	1.12(0.95,1.33)	<0.001	1.05(0.88,1.21)	1.06(0.86,1.26)	0.908
LDL (mmol/L)	3.03(2.47,3.70)	2.97(2.42,3.57)	0.098	3.04(2.48,3.72)	3.03(2.45,3.60)	0.159

**Table 2:** Baseline characteristics and AMI—SCAD risk factors in the study population.

	Full Population			Propensity-Score-Matched Population		
	AMI Patients (N = 702)	SCAD Patients (N = 1253)	*p*	AMI Patients (N = 664)	SCAD Patients (N = 664)	*p*
Age (years)	68.00(60.00,77.00)	65.00(57.00,73.00)	<0.001	68.00(60.00,76.75)	67.00(59.00,76.00)	0.454
Gender			<0.001			0.431
Male (%)	429(61.10%)	657(52.40%)		399(60.10%)	413(62.20%)	
Female (%)	273(38.90%)	596(47.60%)		265(39.90%)	251(37.80%)	
Smokers (Yes/No)	375/327	466/787	<0.001	341/323	340/324	0.956
Hypertension (Yes/No)	361/341	632/621	0.676	340/324	354//310	0.442
Diabetes (Yes/No)	183/519	223/1030	<0.001	163/501	154/510	0.562
TG (mmol/L)	1.50(1.09,2.11)	1.41(1.01,2.04)	0.050	1.50(1.09,2.11)	1.42(1.03,2.03)	0.178
Tc (mmol/L)	4.53(3.87,5.36)	4.69(3.93,5.50)	0.033	4.54(3.88,5.37)	4.64(3.84,5.45)	0.607
HDL (mmol/L)	1.02(0.88,1.21)	1.08(0.91,1.29)	<0.001	1.03(0.89,1.21)	1.02(0.87,1.22)	0.302
LDL (mmol/L)	3.03(2.47,3.70)	3.08(2.47,3.68)	0.629	3.03(2.47,3.69)	3.06(2.43,3.71)	0.686

**Table 3:** Baseline characteristics and AMI + SCAD—control risk factors in the study population.

	Full Population			Propensity-Score-Matched Population		
	AMI + SCAD Patients (N = 1955)	Controls (N = 2288)	*p*	AMI + SCAD Patients (N = 1397)	Controls (N = 1397)	*p*
Age (years)	66.00(58.00,75.00)	56.00(47.00,66.00)	<0.001	63.00(55.00,70.00)	63.00(55.00,70.00)	0.982
Gender			<0.001			0.849
Male (%)	1086(55.50%)	1124(49.10%)		751(53.80%)	756(54.10%)	
Female (%)	869(44.50%)	1164(50.90%)		646(46.20%)	641(45.90%)	
Smokers (Yes/No)	841/1114	736/1552	<0.001	562/835	544/853	0.486
Hypertension (Yes/No)	993/962	994/1294	<0.001	686/711	667/730	0.472
Diabetes (Yes/No)	406/1549	166/2122	<0.001	169/1228	162/1235	0.682
TG (mmol/L)	1.44(1.05,2.07)	1.35(0.96,2.02)	<0.001	1.43(1.03,2.08)	1.41(1.01,2.07)	0.326
Tc (mmol/L)	4.64(3.91,5.41)	4.58(3.91,5.31)	0.246	4.69(3.95,5.42)	4.64(3.94,5.40)	0.792
HDL (mmol/L)	1.06(0.90,1.26)	1.12(0.95,1.33)	<0.001	1.09(0.92,1.29)	1.09(0.92,1.30)	0.724
LDL (mmol/L)	3.05(2.47,3.68)	2.97(2.42,3.57)	0.005	3.07(2.48,3.68)	3.05(2.51,3.69)	0.978

**Table 4:** Association between the levels of metals and AMI—control risk in the multivariable logistic regression model.

	Full Population				Propensity-Score-Matched Population			
	AMI Patients (N = 702)	Controls (N = 2288)	OR (95% CI)	*p*	AMI Patients (N = 586)	Controls (N = 586)	OR (95% CI)	*p*
Na	139.10(137.10,140.90)	139.80(138.20,141.20)	0.980(0.950,1.011)	0.1942	139.15(137.24,140.80)	139.80(138.00,141.20)	0.980(0.940,1.022)	0.353
K	4.01(3.72,4.29)	4.06(3.85,4.28)	1.083(0.870,1.347)	0.4751	4.01(3.74,4.29)	4.08(3.85,4.29)	0.943(0.703,1.266)	0.698
Ca	2.23(2.14,2.31)	2.33(2.25,2.40)	0.049(0.024,0.100)	<0.001	2.24(2.15,2.33)	2.30(2.21,2.37)	0.492(0.186,1.298)	0.152
Mg	0.82(0.76,0.87)	0.81(0.76,0.86)	5.377(2.021,14.309)	0.0008	0.82(0.76,0.87)	0.81(0.76,0.86)	2.758(0.757,10.053)	0.124
Fe	9.70(6.57,13.63)	15.37(10.99,20.36)	0.903(0.888,0.918)	<0.001	9.89(6.50,14.04)	15.04(10.88,19.67)	0.905(0.886,0.925)	<0.001
Zn	13.58(11.80,15.62)	14.59(12.92,16.50)	0.989(0.957,1.022)	0.5212	13.68(11.82,15.69)	14.37(12.80,16.54)	0.992(0.951,1.034)	0.697

**Table 5:** Association between the levels of metals and AMI—SCAD risk in the multivariable logistic regression model.

	Full Population				Propensity-Score-Matched Population			
	AMI Patients (N = 702)	SCAD Patients (N = 1253)	OR (95% CI)	*p*	AMI Patients (N = 664)	SCAD Patients (N = 664)	OR (95% CI)	*p*
Na (mmol/L)	139.10(137.10,140.90)	140.00(138.30,141.40)	0.966(0.937,0.996)	0.0270	139.10(137.15,140.90)	139.90(138.10,141.30)	0.975(0.943,1.009)	0.154
K (mmol/L)	4.01(3.72,4.29)	4.06(3.83,4.31)	0.858(0.682,1.080)	0.1930	4.01(3.72,4.29)	4.05(3.80,4.32)	0.885(0.678,1.155)	0.368
Ca (mmol/L)	2.23(2.14,2.31)	2.31(2.23,2.40)	0.119(0.056,0.256)	<0.001	2.23(2.14,2.31)	2.29(2.20,2.37)	0.333(0.138,0.800)	0.014
Mg (mmol/L)	0.82(0.76,0.87)	0.81(0.77,0.86)	1.855(0.755,4.559)	0.1780	0.82(0.76,0.87)	0.81(0.76,0.86)	1.764(0.660,4.713)	0.258
Fe (mmol/L)	9.70(6.57,13.63)	14.83(10.85,19.51)	0.898(0.881,0.915)	<0.001	9.78(6.60,13.74)	14.11(10.35,18.90)	0.906(0.888,0.925)	<0.001
Zn (mmol/L)	13.58(11.80,15.62)	14.09(12.31,15.86)	1.066(1.029,1.105)	0.0004	13.62(11.83,15.63)	13.89(12.16,15.60)	1.069(1.026,1.114)	0.001

**Table 6:** Association between the levels of metals and AMI + SCAD—controls risk in the multivariable logistic regression model.

	Full Population				Propensity-Score-Matched Population			
	AMI + SCAD Patients (N = 1955)	Controls (N = 2288)	OR (95% CI)	*p*	AMI + SCAD Patients (N = 1397)	Controls (N = 1397)	OR (95% CI)	*p*
Na (mmol/L)	139.70(137.90,141.20)	139.80(138.20,141.20)	0.991(0.970,1.013)	0.4348	139.80(138.10,141.20)	140.00(138.40,141.40)	0.976(0.951,1.003)	0.077
K (mmol/L)	4.05(3.80,4.30)	4.06(3.85,4.28)	1.106(0.948,1.291)	0.2009	4.05(3.81,4.29)	4.07(3.85,4.29)	1.024(0.846,1.239)	0.806
Ca (mmol/L)	2.28(2.19,2.37)	2.33(2.25,2.40)	0.260(0.161,0.419)	<0.001	2.30(2.21,2.39)	2.32(2.24,2.39)	1.036(0.582,1.841)	0.905
Mg (mmol/L)	0.82(0.76,0.86)	0.81(0.76,0.86)	2.921(1.490,5.727)	0.0018	0.81(0.77,0.86)	0.81(0.76,0.86)	1.799(0.822,3.938)	0.142
Fe (mmol/L)	12.98(8.80,,18.10)	15.37(10.99,20.36)	0.968(0.959,0.977)	<0.001	13.74(9.31,18.71)	15.56(11.30,20.32)	0.968(0.957,0.979)	<0.001
Zn (mmol/L)	13.93(12.10,15.75)	14.59(12.92,16.50)	0.952(0.931,0.974)	<0.001	14.14(12.27,15.97)	14.42(12.77,16.38)	0.975(0.949,1.002)	0.069

**Table 7:** The ROC curve of the AMI vs. the SCAD group.

AMI vs. Control in Full Population	AUC	AMI vs. Control in Propensity-Score-Matched Population	AUC
Na	0.578	Na	0.561
K	0.530	K	0.537
Ca	0.699	Ca	0.618
Mg	0.489	Mg	0.484
Fe	0.724	Fe	0.702
Zn	0.598	Zn	0.573

**Table 8:** The ROC curve of the AMI + SCAD vs. the control group.

AMI vs. SCAD in Full Population	AUC	AMI V SCAD in Propensity-Score-Matched Population	AUC
Na	0.584	Na	0.567
K	0.535	K	0.526
Ca	0.666	Ca	0.613
Mg	0.499	Mg	0.488
Fe	0.710	Fe	0.682
Zn	0.542	Zn	0.519
